# Supplementary material for: Unraveling the associations between voice pitch and major depressive disorder: a multisite genetic study
Source: Mol Psychiatry. 2024 Dec 31;30(6):2686–95. doi: 10.1038/s41380-024-02877-y (PMC12092242; doi:10.1038/s41380-024-02877-y)
Supplement: Supplementary file 1 — Supplemental material [file 41380_2024_2877_MOESM1_ESM.docx]

Supplementary materials for:

**Unraveling the Associations Between Voice Pitch and Major Depressive Disorder: A Multisite Genetic Study**

Yazheng Di ^1,2^, Elior Rahmani^3^, Joel Mefford^4^, Jinhan Wang^5^, Vijay Ravi^5^,., Aditya Gorla^6^, Abeer Alwan^5^, Kenneth S. Kendler^7^,^8^ Tingshao Zhu^1,2^, Jonathan Flint^9^.

1.CAS Key Laboratory of Behavioral Science, Institute of Psychology, Beijing 100101, China.

2.Department of Psychology, University of Chinese Academy of Sciences, Beijing 100049, China.

3.Department of Computational Medicine, University of California Los Angeles, Los Angeles, CA, USA.

4.Department of Neurology, University of California Los Angeles, Los Angeles, CA, USA.

5.Department of Electrical and Computer Engineering, University of California Los Angeles, Los Angeles, CA, USA.

6.Bioinformatics Interdepartmental Program, University of California Los Angeles, Los Angeles, CA, USA

7.Virginia Institute for Psychiatric and Behavioral Genetics, Richmond, VA, USA

8.Department of Psychiatry, Virginia Commonwealth University School of Medicine, Richmond, VA, USA

9.Department of Psychiatry and Biobehavioral Sciences, Brain Research Institute, University of California Los Angeles, Los Angeles, CA, USA.

The Supplementary contained the following contents:

- Supplementary Methods
- Figure S1-S11
- Tables S1-S10 (in a separate MS Excel file)

**Supplementary Results**

*Analysis on accent (Mandarin vs. non-Mandarin)*

We carried out within-group analyses (of Mandarin speakers and non-Mandarin speakers) and compared the results by using Cochran’s Q test (1) for heterogeneity. The results are shown in the following **Table S9** and **Figure S10**. We found that there was no significant heterogeneity in the association effects between Mandarin speakers and non-Mandarin speakers. The lowest heterogeneity p value is 0.047 (for ΔF0_quartile3). This is the only P value that is <0.05 for heterogeneity test on the 20 features (after applying a multiple-test correction this result is not significant at a 5% level).

*Analysis on audio concatenation*

The extracted features were from the concatenation of the audio segments from patients. We tested whether the concatenation introduced bias by comparing results carried out on features extracted from an individual segment with those from the concatenated results.

For each participant, we selected the longest utterance, extracted features, and repeated the meta-analysis. The results of the longest segment analysis are shown in **Figure S11**, comparing the analysis of concatenated speech and longest segments. Pearson’s correlation of the beta values between the two methods is high: r = 0.9, p = 1.9${\times10}^{-9}$.

Restricting analysis to the longest segments however loses power for some features, as we are removing considerable amounts of information from each recording (on average 171.76 seconds (SD=198.94)).

*Genome-wide association analysis (GWAS) on the heritable voice features*

We performed GWAS on the 7,654 subjects in CONVERGE (**Figure S1**) for the four heritable features in **Table 3**: ΔF0_iqr1-3, F0_kurtosis, ΔF0_kurtosis, and ΔF0_percentile99.0. We did not find any significant hits, presumably owing to the limited sample size. The Manhattan plots and Q-Q plots are in **Figure S12.** SNPs associated with corresponding features at a P-value ${<10}^{-5}$ are listed in **Table S10**.

GWAS Summary Statistics are available at <https://doi.org/10.6084/m9.figshare.24571321.v1>).

*Comparison between feature-based and network-based models.*

An alternative to testing all features (and hence to avoid a multiple testing burden) is to turn to neural network-based approaches (Convolutional Neural Networks and Recurrent Neural Networks). **Table S11** compares our previous work (2) based on audio neural networks and current features-based methods. We found that the performance of the network-based method in our replication sample drops below that of the feature-based. We suspect this is because of the complex set of confounds that we deal with using the meta-analytic approach.

**Supplementary Methods**

*Interview protocol*

All subjects were interviewed using a computerized assessment system. Interviewers were postgraduate medical students, junior psychiatrists, or senior nurses, trained by the CONVERGE team for a minimum of 1 week. Interviews were recorded and the research team listened to at least two interviews from each interviewer to identify any errors in the way questions were asked and answers were interpreted. The interview protocol acquired the following assessments for psychopathology: i) CIDI (WHO 1997) section of MDD expanded to include a “deep” assessment of the DSM-IV A criteria for MDD, symptoms of DSM-IV melancholia, Beck’s cognitive triad (helplessness, hopefulness, and worthlessness), and irritability/anxiety; ii) CIDI section on dysthymia; iii) sections from interviews in the Virginia Adult Twin Study of Psychiatric and Substance Use Disorders (VATSPSUD) (3) for generalized anxiety disorder, panic, and five phobia subtypes (agoraphobia and social, situational, animal, and blood injury phobias), (iv) brief assessments of premenstrual syndrome and postnatal depression (4, 5), and (v) assessments of smoking/nicotine dependence (alcohol and substance abuse were virtually absent in this study, so it was not assessed). Additionally, four key environmental exposures known to be strongly associated with the risk of MDD were assessed in cases and controls: i) child sexual abuse; ii) parent-child relationships; iii) social support; and iv) stressful live events. Neuroticism was assessed using the full 23-item Eysenck personality questionnaire N scale. Family history of MDD was individually assessed in parents and full siblings using the Family History Research Diagnostic Criteria. In each case, measures used are those developed, field-tested, and validated in the VATSPSUD studies (5).

The interview protocol in the replication study mirrored that used in the CONVERGE study, with the addition of a 16-item, self-administered questionnaire assessing the severity of depression-related symptoms on a five-point distress scale over the past 30 days (subscales for depression in symptom checklist, SCL).

## *DNA Sequencing and Genotype imputation*

The CONVERGE study used low-coverage sequencing to genotype the sample. DNA was extracted from saliva samples using the Oragene protocol. Sequencing reads obtained from Illumina Hiseq machines were aligned to Genome Reference Consortium Human Build 37 patch release 5 (GRCh37.p5) with Stampy (v1.0.17)(6) using default parameters after filtering out reads containing adaptor sequences or consisting of more than 50% poor quality bases. The aligned reads were indexed, and PCR duplicates were marked for removal. Base quality score recalibration (BQSR) was performed on the BAM files using the Genome Analysis Toolkit (GATK) (7), with known SNPs and INDELs masked. Whole-genome sequences were acquired to a mean depth of 1.73 (95% confidence intervals (CIs) 0.7–4.3) per individual, from which 32,781,340 SNP sites were identified.

Variant discovery and genotyping were conducted using the GATK's UnifiedGenotyper, targeting polymorphic SNPs in the 1000 Genomes Project Phase 1 East Asian reference panel. The dbSNP v137 rsids were used to fill in the variant ID column of the output variant call format (VCF) files. A sensitivity threshold of 90% to SNPs in the 1000G Phase1 ASN panel was applied for SNP selection for imputation. This gave a total of 21,356,798 (9,053,391 known in 1000 Genomes Phase 1 ASN Panel and 11,486,024 novel) biallelic SNPs identified from all chromosomes and unassembled contigs. Genotype likelihoods were calculated using SNPtools (8). The imputation process was conducted using BEAGLE software (9).

*Feature selection process to remove redundant features*

From the COMPARE16 feature set, we obtained 83 F0/ΔF0-based features. We excluded one feature, the minimal length for F0 >0 because the values of this feature were the same for almost all subjects. They were forced to the frame size unit, according to reference (10). Next, given that many of the features were highly correlated (for example, the arithmetic and root-quadratic mean of F0) (**Figure S2**), we implemented a feature selection process to remove redundant features. We first calculated the pairwise Pearson correlation for each voice feature pair and ranked the voice features from high to low according to their sum of squared correlation r values with all other features. We then retained the first (leading) feature and excluded all other features that had an absolute r value >0.5 with the leading one. This process was repeated, each time keeping the next leading feature remaining in the list and excluding its correlates. The above procedure resulted in a set of 30 F0/ΔF0-based features (**Table S2**) that were representative of the original 82 features and not highly correlated with each other.

*Genome-Wide Association Studies (GWAS)*

We performed GWAS for each one of the heritable voice features on the 7,654 subjects utilizing the LDAK tool (11). A genetic relationship matrix (GRM), constructed from the genotype dataset, was utilized to correct for relatedness among the samples. We applied rank-based inverse normal transformation to the voice features and incorporated these covariates into our analysis: 20 genetic PCs, age, education level, occupation, marital status, social class, noise level, and accent.

**Reference**

1. Cochran WG: The combination of estimates from different experiments. Biometrics 1954; 10:101–129

2. Wang J, Ravi V, Flint J, et al.: Speechformer-CTC: Sequential modeling of depression detection with speech temporal classification. Speech Commun 2024; 163:103106

3. Kendler KS, Prescott CA: Genes, environment, and psychopathology: Understanding the causes of psychiatric and substance use disorders. Guilford Press, 2007

4. Cox JL, Holden JM, Sagovsky R: Detection of postnatal depression: development of the 10-item Edinburgh Postnatal Depression Scale. Br J Psychiatry 1987; 150:782–786

5. Kendler K, Silberg J, Neale M, et al.: Genetic and environmental factors in the aetiology of menstrual, premenstrual and neurotic symptoms: a population-based twin study. Psychol Med 1992; 22:85–100

6. Lunter G, Goodson M: Stampy: a statistical algorithm for sensitive and fast mapping of Illumina sequence reads. Genome Res 2011; 21:936–939

7. McKenna A, Hanna M, Banks E, et al.: The Genome Analysis Toolkit: a MapReduce framework for analyzing next-generation DNA sequencing data. Genome Res 2010; 20:1297–1303

8. Wang Y, Lu J, Yu J, et al.: An integrative variant analysis pipeline for accurate genotype/haplotype inference in population NGS data. Genome Res 2013; 23:833–842

9. Browning SR, Browning BL: Rapid and Accurate Haplotype Phasing and Missing-Data Inference for Whole-Genome Association Studies By Use of Localized Haplotype Clustering. Am J Hum Genet 2007; 81:1084–1097

10. Eyben F: Real-time speech and music classification by large audio feature space extraction. Springer, 2015

11. Speed D, Cai N, Johnson MR, et al.: Reevaluation of SNP heritability in complex human traits. Nat Genet 2017; 49:986–992

**Supplementary Figures**

**Figure S1. Study overview.** a) study aims and main analyses. QC: quality control. The criteria of inclusion of sites for meta-analysis is a sample size N > 100, and a case/controls ratio between 0.1 and 0.9. b) PRISMA diagram of the quality control process and selection of sites for meta-analysis.


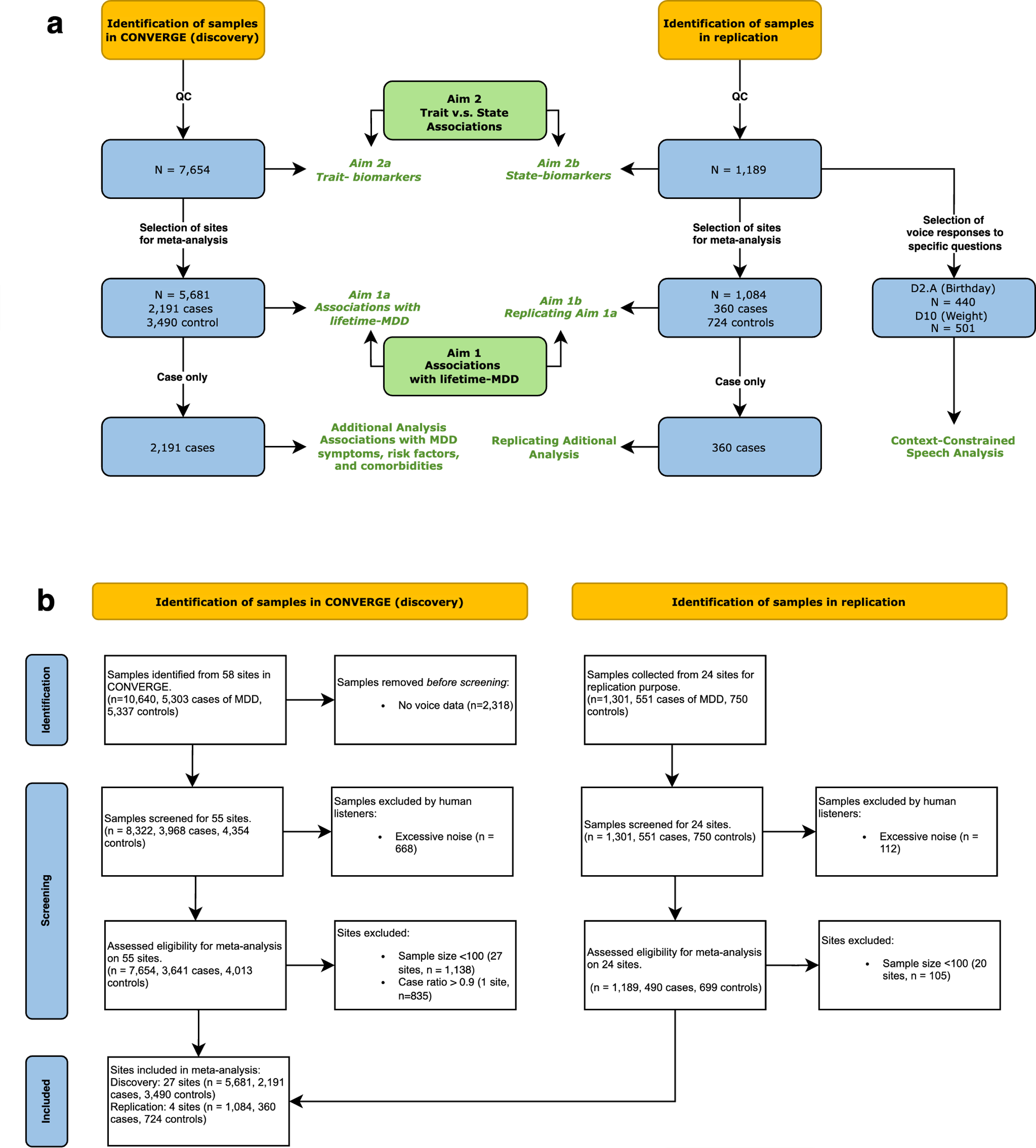


**Figure S2. Heatmap of the correlation r-values between the leading features and excluded features.**

The x-axis shows the 52 voice features excluded because of their high correlation with the 30 leading features (y-axis).

**
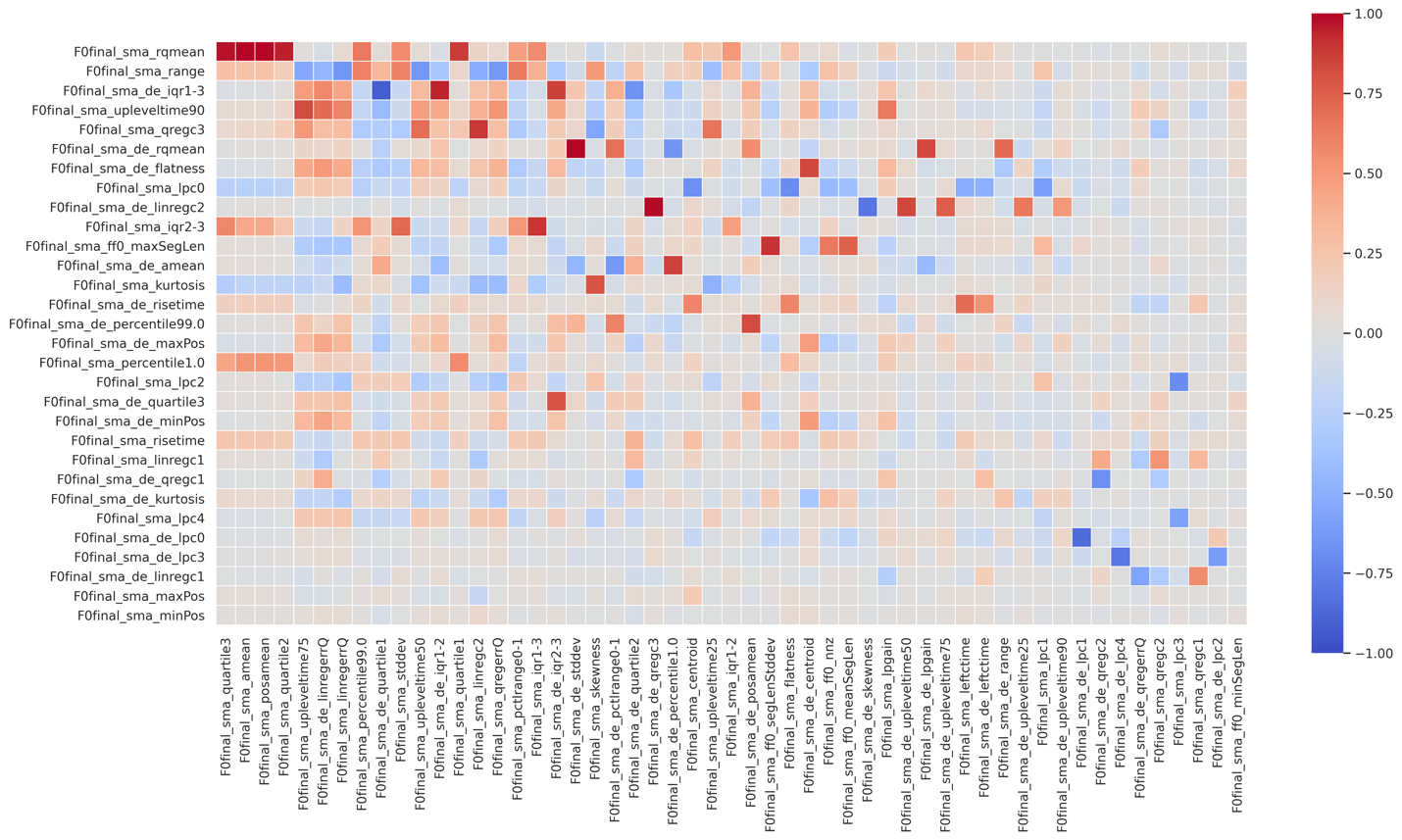
**

**Figure S3. Distribution of the 30 pitch features (original values).**


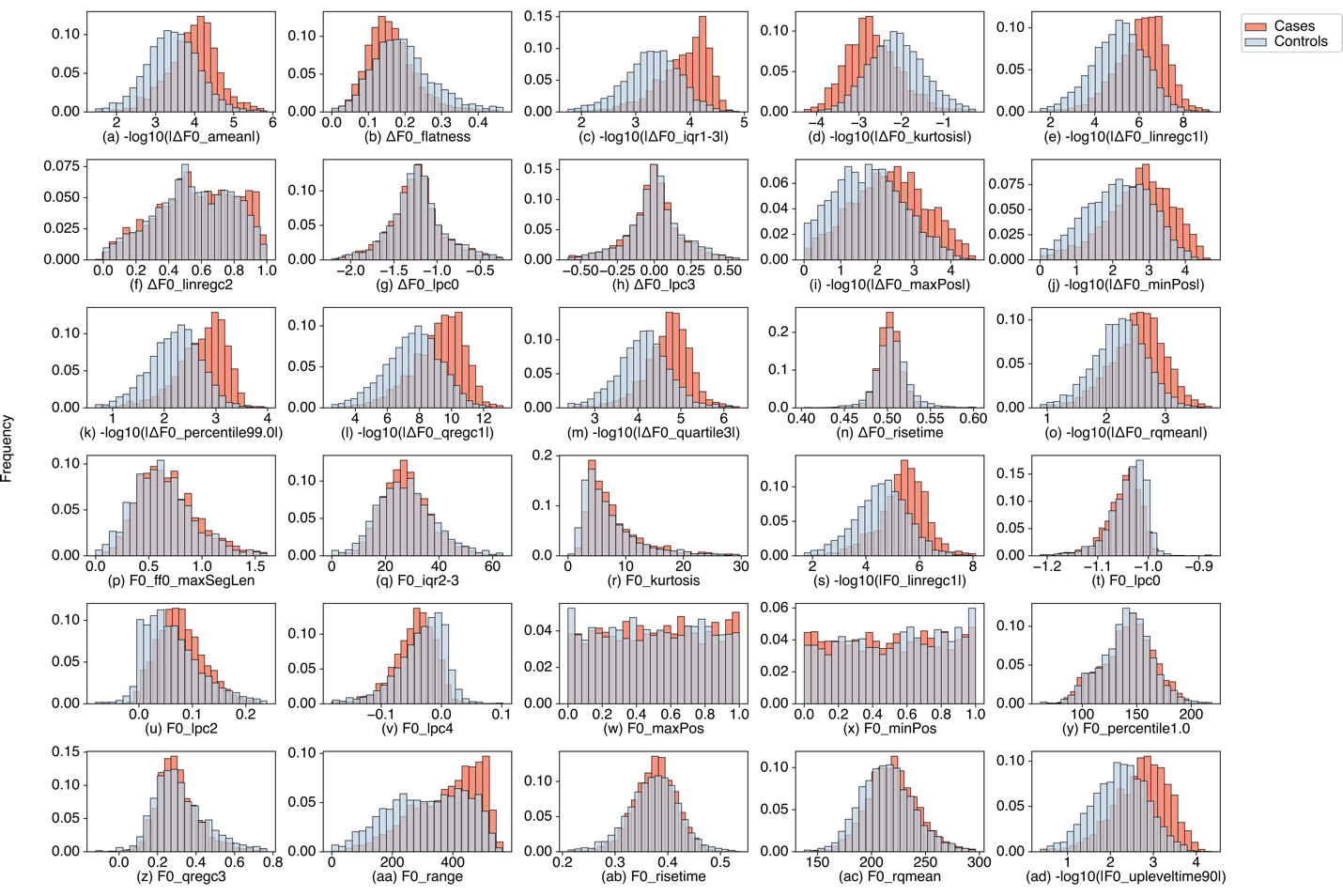


**Figure S4. Distribution of the 30 pitch features after normalization.**


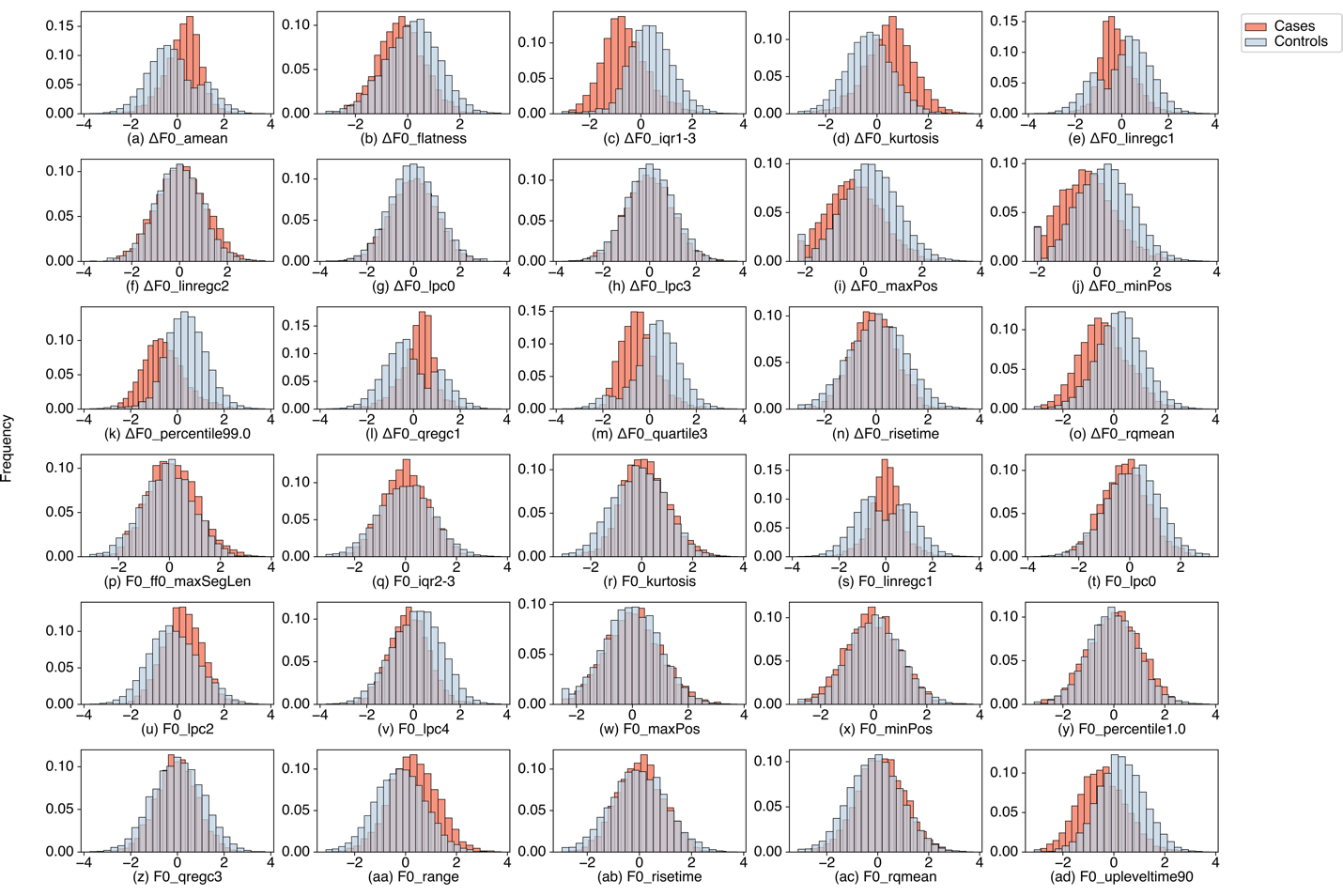


**Figure S5**. **Results of the meta-analysis with and without adjusting for genetic PCs.**

**
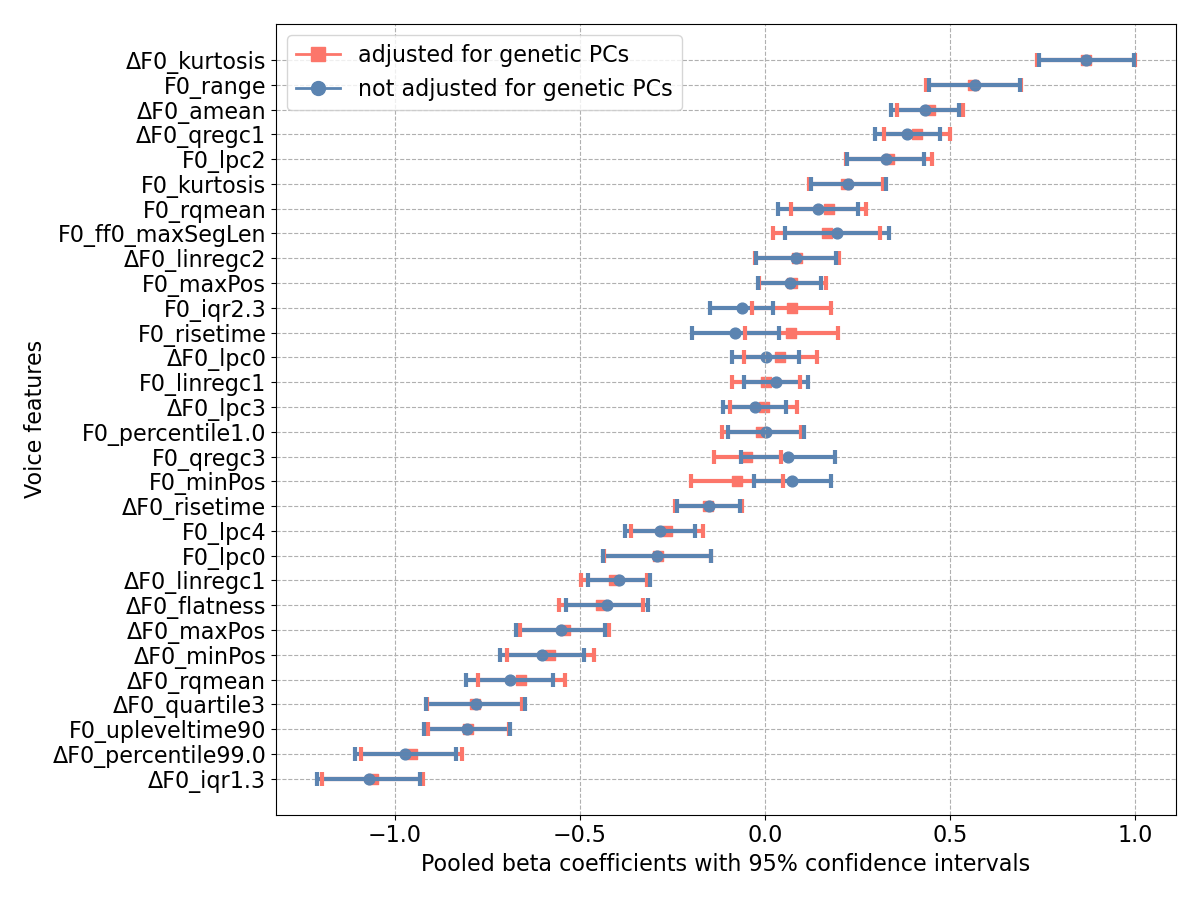
**

**Figure S6. Estimated beta coefficients with 95% confidence interval from associations between 16 voice F0/ΔF0 features and MDD.**

Estimations were conducted using the two-stage meta-analysis method on four cohorts: CONVERGE, replication, single-segment about weight (D10) from replication, and single-segment about birthday (D2.A) from replication.


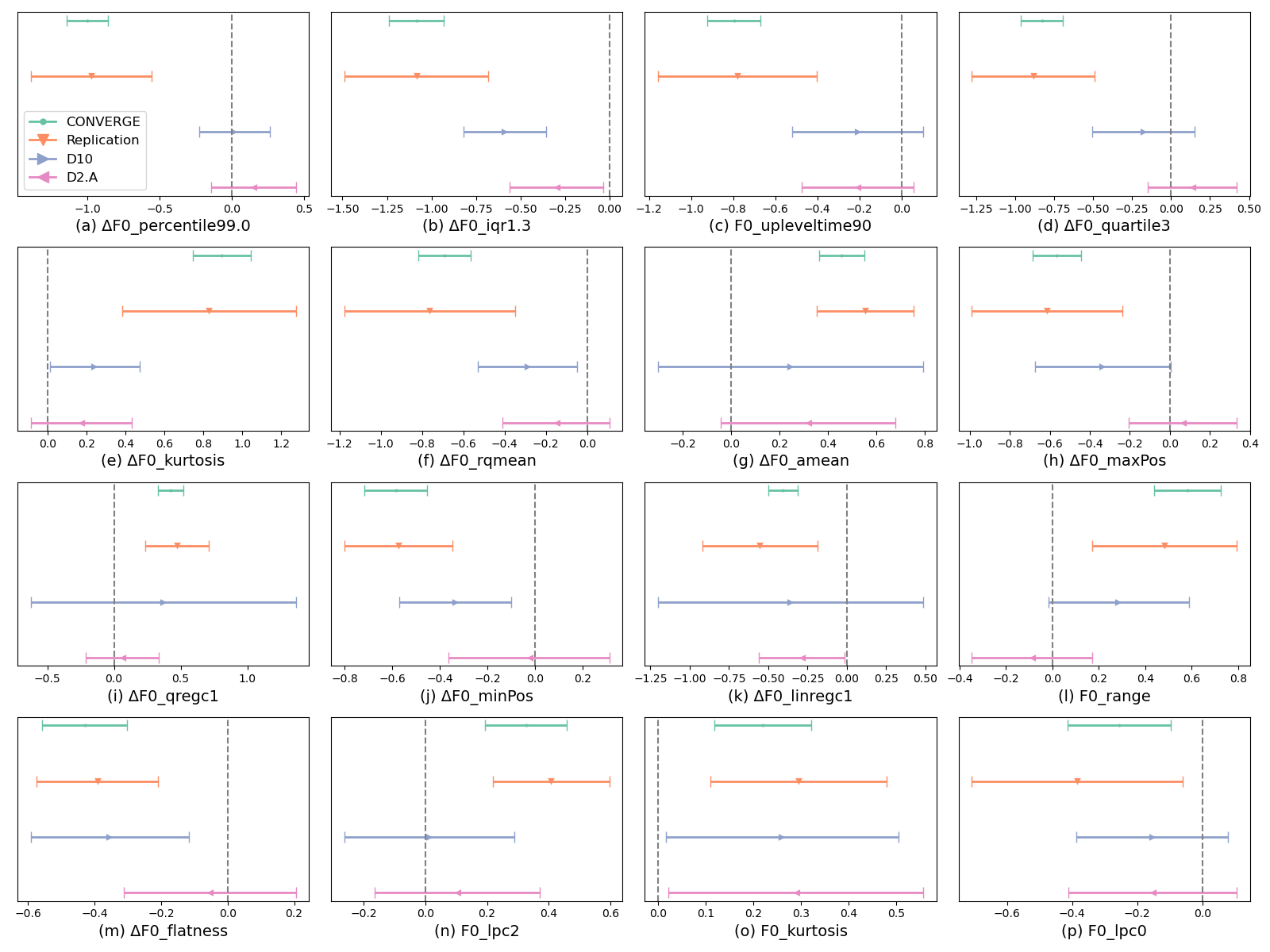


**Figure S7. The distribution of the Symptom Checklist (SCL) scores in MDD cases and controls.**

**
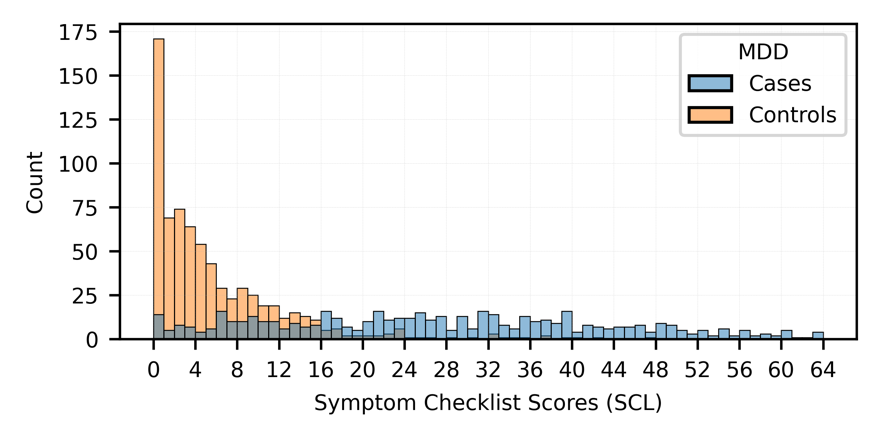
**

**Figure S8. Precision-recall curve of classification performance on test (replication) samples.** The figure shows the precison-recall curve for four models for predicting depression from voice features, and a null model, a logistic regression model trained on demographic covariates only (LR-Covar). The full models are logistic regression (LR), support vector machine (SVM), multi-layer perceptron (MLP), and extreme gradient boosting (XGBoost), trained on voice and covariates (Covar+Voice). AP: average precision score.

*
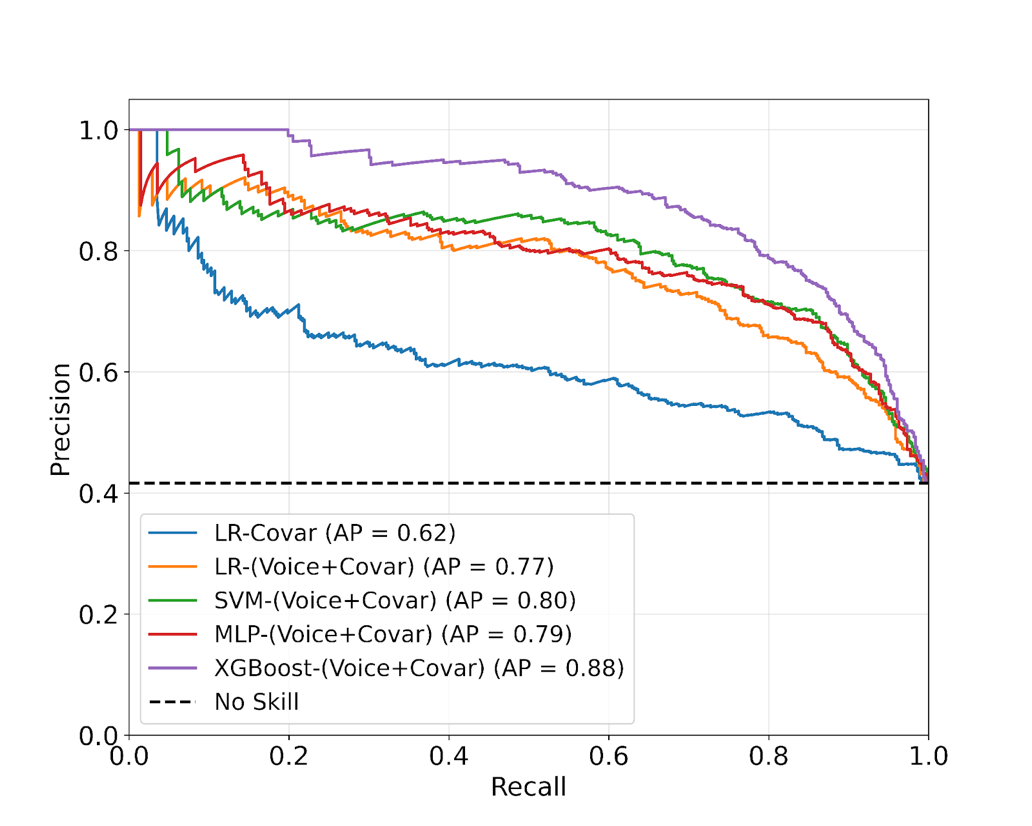
*

**Figure S9.** **Distribution of the audio segment length.**

**
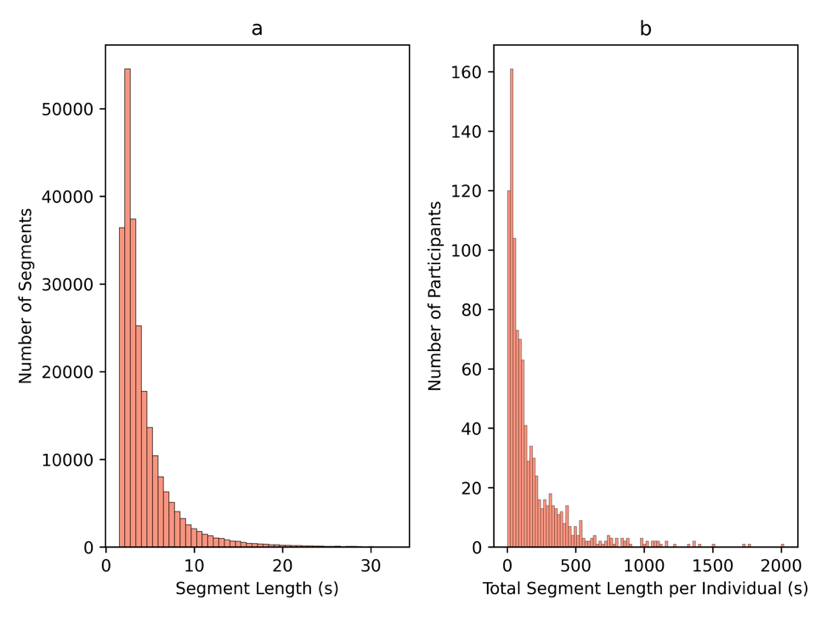
**

**Figure S10. Results of the meta-analysis on Mandarin and non-Mandarin speakers.**


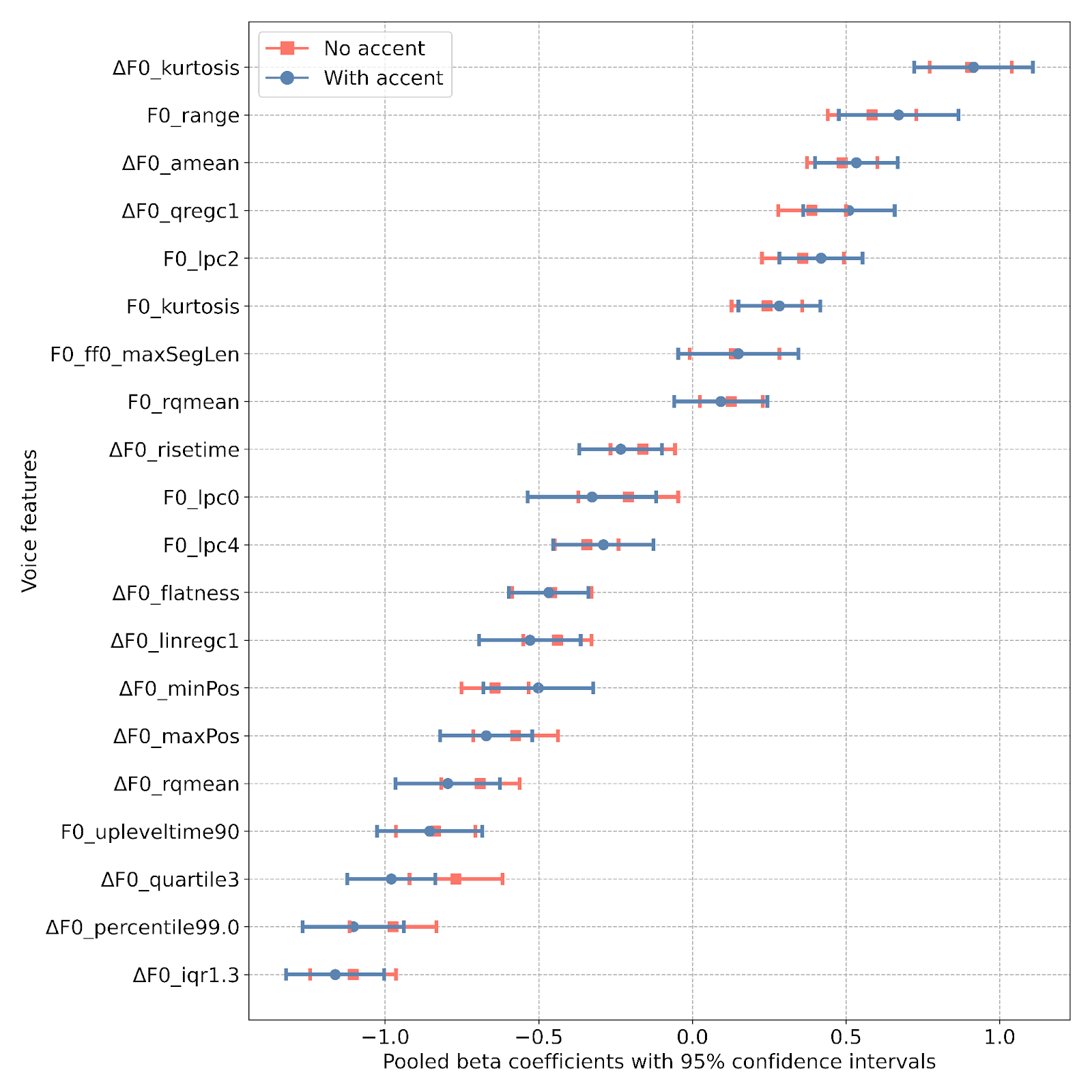


**Figure S11. Results of meta-analysis for single longest audio segments and concatenated speech.** Concatenated Speech: This is the original meta-analysis we have reported in Table 1, where for each individual, the voice features were extracted from the concatenated segments. Longest Segment: For each individual, their longest segment was used to extract the features. Then we repeated the same meta-analysis procedure as we did in the original.


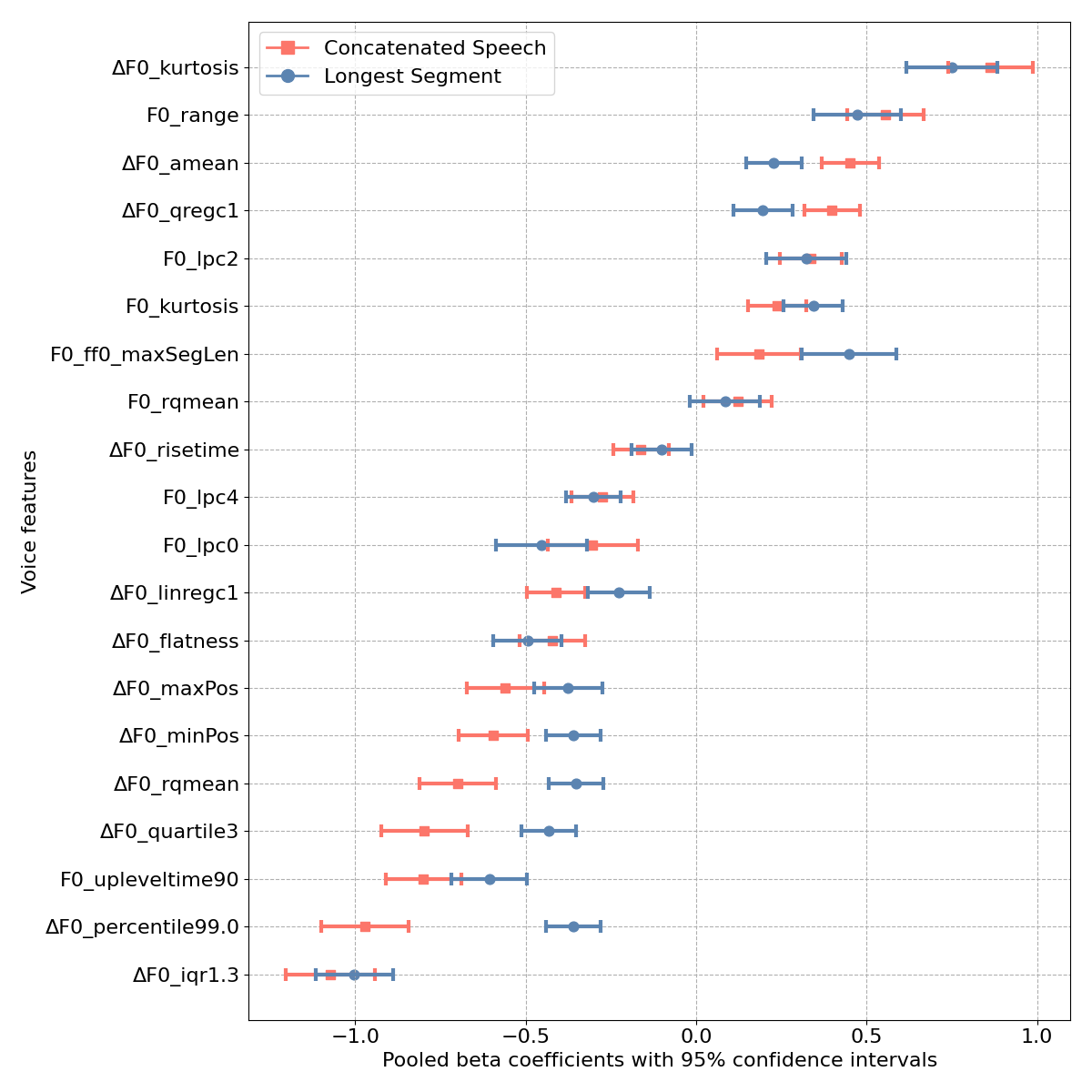


**Figure S12. GWAS results for a) ΔF0_iqr1-3, b) ΔF0_kurtosis, c) ΔF0_percentile99.0, and d) F0_kurtosis.**

**
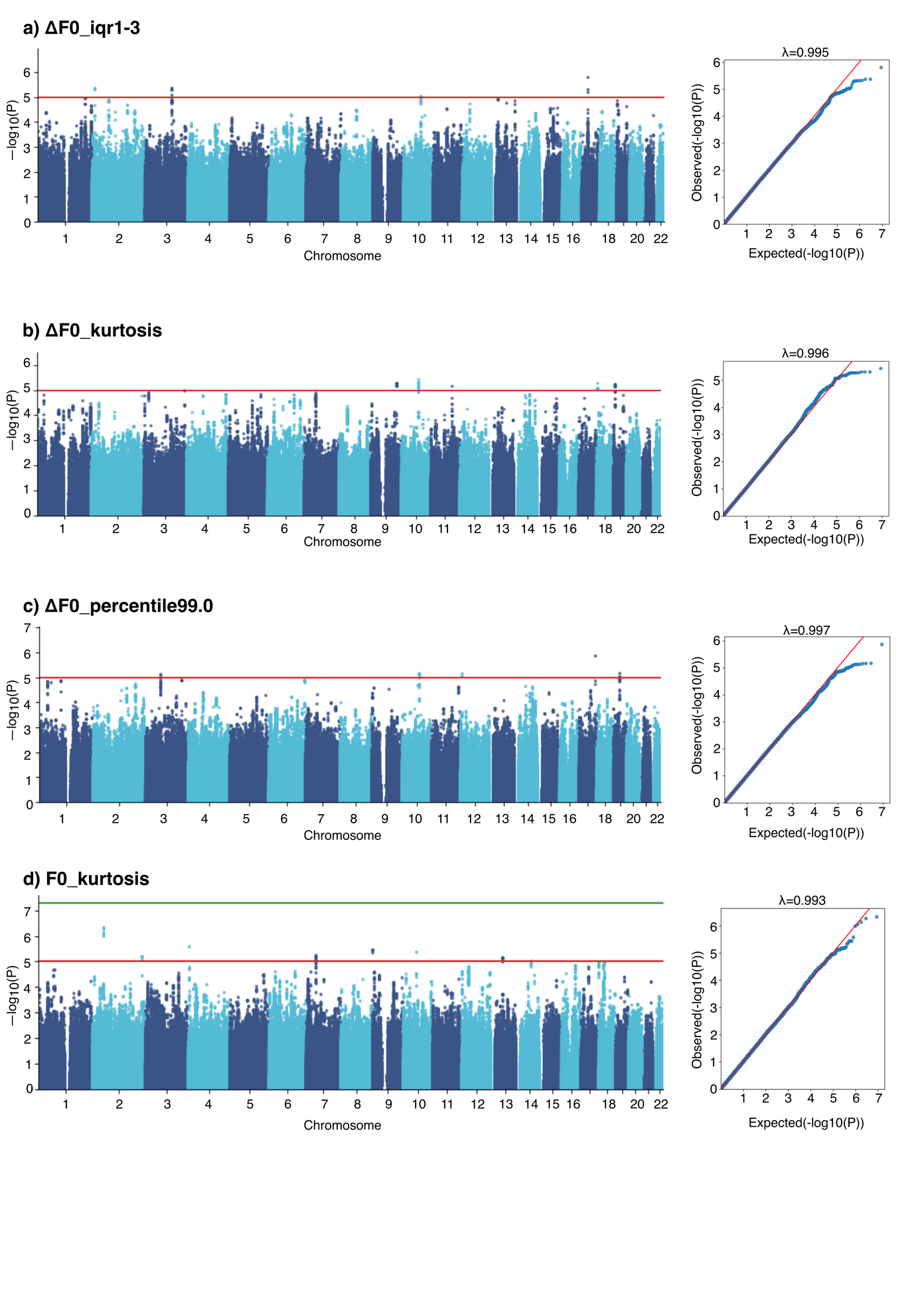
**
